# AI for dental imaging: impressive in vitro, but what about in practice?

**DOI:** 10.1038/s41432-025-01198-5

**Published:** 2025-12-04

**Authors:** Thomas Evans, Henry David Jeffry Hogg

**Affiliations:** 1 AI and Digital Health Team, University of Birmingham, Birmingham, United Kingdom; 2 Special Care Dental Services, Birmingham Community NHS Trust, Birmingham, United Kingdom; 3 University of Birmingham, Birmingham, United Kingdom; 4 University Hospitals Birmingham NHSFT, Birmingham, United Kingdom

**Keywords:** Dental radiology, Dentistry

## Abstract

**A Commentary on:**

**Alam M K, Alftaikhah S A A, Issrani R** **et al**.

Applications of artificial intelligence in the utilisation of imaging modalities in dentistry: a systematic review and meta-analysis of in-vitro studies. *Heliyon* 2024; 10.1016/j.heliyon.2024.e24221.

**Design:**

A systematic review of in vitro studies utilising artificial intelligence (AI) in dental imaging. Searches were carried out across multiple databases: CINAHL, Cochrane Library, Embase, Google Scholar, IEEE Xplore, PubMed/MEDLINE, Scopus, and Web of Science as well as hand-searching references from eligible articles.

**Study selection:**

Studies were eligible if i) classed as in vitro, defined as simulations or laboratory tests outside a living organism, ii) studied the performance of AI techniques and iii) involved analysis of dental imaging. Studies not in English or with insufficient data were excluded.

**Data analysis:**

An adapted version of the CONSORT bias tool was used for the assessment of studies. Outcome measures were extracted including: odds ratios, true positive rate, true negative rate, positive predictive value, and negative predictive value. A meta-analysis using a fixed-effects model assessed accuracy with a 95% confidence interval. Heterogeneity and overall effect tests were applied to evaluate the reliability of the meta-analysis.

**Results:**

After screening, nine studies were identified, eight of which focused on Cone Beam Computed Tomography (CBCT) imaging. Endpoints included caries detection, segmentation tasks and virtual 3D model creation. Across the nine studies, and when pooled in meta-analysis, AI performance was shown to be superior to reference standards.

**Conclusions:**

This systematic review of in-vitro studies highlights the potential of AI to improve the speed or quality of dental imaging tasks. However clinical studies are required to ensure evidence from laboratory studies can translate into clinical practice.

## GRADE Rating:



## Commentary

Artificial intelligence (AI) use in radiology has been growing, especially since deep learning has been shown to surpass humans in some specific use cases. Alam et al.’s systematic review^1^ focuses on in vitro studies of AI as applied to imaging in dentistry. The purpose of this study was to examine AI image analysis techniques against reference standards or human performance in controlled settings. A variety of clinical tasks were identified including segmentation and classification of teeth, segmentation of bone, caries detection, multiple disease diagnosis and creation of virtual 3D models. Non-interventional studies are a valuable step in the pathway for clinical innovations, often presenting a proof-of-concept validation, whilst circumventing the clinical risk and regulatory oversight associated with interventional studies.

Initial searches returned 617 potentially eligible records, but only nine in vitro studies were ultimately found eligible. The focus on in vitro may have unintentionally excluded many important studies, as many non-clinical validation studies may not been identified by the search strategy and the term is lesser used in the field of AI evaluation.

In the nine studies identified eight utilised CBCT and one panoramic radiographs. The modalities used in the studies identified also don’t represent most of the dental imaging undertaken in general practice - but may still be of relevance for those using CBCT. The AI models identified by this review do show superiority over the reference standards assessed. Different outcome measures were used across the studies, most commonly reflecting speed of analysis, diagnostic accuracy or both. The AI systems were compared against standard of care approaches, or clinicians replicating the task the AI was intended for. Furthermore, this systematic review groups together many different forms of machine learning model types including deep learning, convolutional neural networks and combined models. Taken in combination with the low number of studies and study heterogeneity, the meta-analysis reported is of limited utility.

AI specific reporting guidelines, or extensions are available for many different types of study, however some were published after the included studies were published and gaps in AI-specific guidelines for different study types remain^2,3^. Whilst using the 2020 PRISMA reporting guidelines the authors also used an adapted CONSORT bias tool, this was originally designed for randomised control trials. An alternative would have been to use a generic bias assessment tool for the study type where AI-specific tools are not yet available, e.g. QUADAS 2, or adapt tools tailored to AI studies, e.g. PROBAST-AI^4–6^.

In vitro studies are limited by their design, and for assessment of patient impact, clinical studies are usually required. This is particularly pertinent for AI systems because they can have large drops in accuracy when exposed to real world context; causes include a different population, a different scanner, or a drift in the practice of health professionals^7^. Furthermore, the studies mainly assessed the direct output of the AI, as opposed to a human using the output. In real-world use the human-computer interaction has a substantial, variable and unstable effect on the effectiveness of AI systems, something which is key to evaluate prior to implementation. The focus on studies done in controlled conditions for diagnostic image analysis is stated as a strength of this study by the authors but is also a weakness in generalisability. An alternative non-interventional methodology would be a “silent-trial”, where an AI model is run in parallel to clinical practice. This has the benefit of being prospective and subject to some of the sources of performance reduction associated with real-world use, but could have been missed as not in vitro^8^. Many of these shortcomings are however pointed out by the authors including the limitations of generalisability to clinical practice.

AI research has a long history; however, clinical integration across clinical specialties remains limited^9^. In and outside of dentistry, the key question for evidence-based clinical practice remains; does the technology have a demonstrable benefit to patient care that outweighs any harms? Whilst this non-interventional evidence of AI surpassing humans in dental image analysis tasks is a promising step toward AI innovation, we must continue to await the evidence that will allow responsible decision-making on whether to adopt these technologies or not.

Practice points

- AI can surpass humans on several aspects of dental image analysis in a laboratory setting.
- Clinical studies are required to demonstrate AI robustness and utility for dental patients.
